# Quenched Zwitterionic Cyclic Arg-Gly-Asp-Containing Pentapeptide Probe for Real-Time Brain Tumor Imaging

**DOI:** 10.3390/pharmaceutics16081034

**Published:** 2024-08-02

**Authors:** Hyunjin Kim, Maixian Liu, Yongdoo Choi

**Affiliations:** Division of Technology Convergence, National Cancer Center, 323 Ilsan-ro, Goyang 10408, Republic of Korea; nalvi77@ncc.re.kr (H.K.); 77200@ncc.re.kr (M.L.)

**Keywords:** activatable probe, zwitterionic, near-infrared fluorescence, brain cancer, imaging

## Abstract

The efficacy of glioblastoma treatment is closely associated with complete tumor resection. However, conventional surgical techniques often result in incomplete removal, leading to poor prognosis. A major challenge is the accurate delineation of tumor margins from healthy tissues. Imaging-guided surgery, particularly using fluorescent probes, is a promising solution for intraoperative guidance. The recently developed ‘always-on’ types of targeted fluorescence probes generate signals irrespective of their presence in tumor cells or in blood circulation, hampering their effectiveness. Here, we propose a novel activatable fluorescence imaging probe, Q-cRGD, that targets glioma cells via the specific binding of the cyclic Arg-Gly Asp-containing pentapeptide (cRGD) to integrins. The Q-cRGD probe was synthesized by conjugating a near-infrared (NIR) dye to a tryptophan quencher via a disulfide linkage, including a cRGD-targeting ligand. This activatable probe remained inactive until the redox-responsive cleavage of the disulfide linkage occurred within the target cell. The zwitterionic nature of NIR dyes minimizes nonspecific interactions with serum proteins, thereby enhancing the tumor-to-background signal ratio (TBR). An in vivo fluorescence imaging study demonstrated a TBR value of 2.65 within 3 h of the intravenous injection of Q-cRGD, confirming its potential utility in imaging-guided brain cancer surgery.

## 1. Introduction

Glioblastoma multiforme (GMF) is one of the most aggressive cancers affecting adults [1]. Although the surgical removal of tumor tissue is currently the most effective way to slow disease progression, the prognosis remains poor, with only rare cases of patients surviving for more than 5 years [2,3]. Relying on subjective palpation and visual identification can lead to incomplete removal and subsequent tumor recurrence [4]. The key to completely removing a brain tumor is the ability to clearly distinguish between healthy and cancerous tissues. Preoperative imaging of malignant tissues shows promise in helping surgeons differentiate between tumors and healthy tissues during surgery. However, craniotomy-induced changes such as cerebrospinal fluid loss complicate the alignment of preoperative images under intraoperative guidance [5].

Recently, intraoperative optical imaging has become an important tool to assist surgeons in the complete removal of tumor tissues. U.S. Food and Drug Administration-approved dye molecules such as fluorescein, 5-aminolevulinic acid (5-ALA), and indocyanine green (ICG) are used in intracranial surgery to remove gliomas [6]. However, these fluorescent agents have limitations, such as low tissue penetration of fluorescence emission, autofluorescence from surrounding normal tissues, or nonspecific accumulation of the injected dye outside the lesions, making it challenging to accurately distinguish between tumors and normal brain tissue [7,8,9]. Compared with passive targeting, contrast agents with targeting abilities are more suitable for precise tumor detection. Antibody-based optical contrast agents show promise for actively targeting cancer cells within tumors. However, the widespread application of antibody–dye conjugates is limited by their long half-life in the bloodstream, which generates high background noise over a prolonged period [10]. The immunochemical staining of glioblastoma has revealed a high expression of various integrins that regulate cancer metastasis and angiogenesis, including αvβ3, αvβ5, αvβ8, and α6β1, which can be targeted by cyclic Arg–Gly–Asp-containing pentapeptide (cRGD) [11,12]. Previously reported fluorescent dye–RGD conjugates successfully targeted integrins in tumors in xenograft and orthotopic animal models [8,13,14]. However, these ‘always-on’ types of targeted fluorescence probes generate signals irrespective of their presence in tumor cells or blood circulation, producing high background noise. The high background noise caused by the always-on mode and nonspecific binding with serum proteins necessitates a long waiting time to build good contrast for visualizing tumor tissue, and brain surgery typically lasts for about 4 h [15].

One strategy to overcome these challenges is to design targeting agents with activatable features [16]. This approach ensures that the imaging probe generates fluorescent signals only when the quencher and fluorescent agents are separated by a biochemical reaction in the target cells, thereby significantly reducing the waiting period after probe injection. Another approach involves the design of fluorescent dye conjugates with zwitterionic features. The targeting moiety promotes the accumulation of the fluorescent dye conjugate in the tumor, whereas the zwitterionic moiety ensures ultralow serum binding, negligible nonspecific tissue background, and rapid elimination through urinary excretion [17,18,19,20].

**Figure 1:**
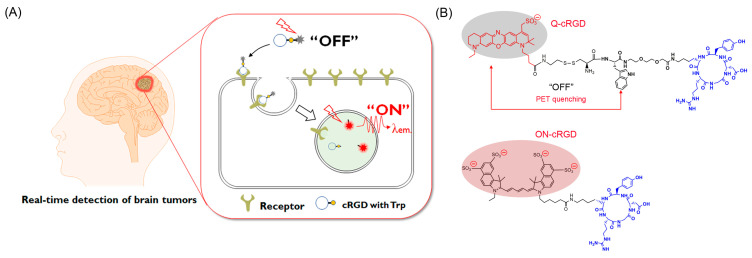
(**A**) Schematic of specific brain tumor imaging using quenched zwitterionic cRGD probe (Q-cRGD). Near-infrared (NIR) fluorescence of Q-cRGD is quenched in blood circulation but its fluorescence is turned on inside target brain tumor cells, leading to therapy enabling specific and real-time fluorescence detection of brain tumors. (**B**) Chemical structures of Q- and ON-cRGD. Q-cRGD consists of zwitterionic NIR fluorescence dye ATTO680, redox cleavable linker, tryptophan as photoinduced electron transfer (PET) quencher, and targeting ligand cRGD. Always-on Cy5.5 dye is conjugated with cRGD in ON-cRGD probe.

In this study, we propose a novel activatable fluorescence imaging probe, Q-cRGD, that targets glioma cells via the specific binding of the cyclic Arg–Gly–Asp-containing pentapeptide (cRGD) to integrins (Figure 1). The Q-cRGD probe was synthesized by conjugating a near-infrared (NIR) dye with a tryptophan (Trp) quencher via a disulfide linkage and a cRGD-targeting ligand. This activatable probe remained inactive until the redox-responsive cleavage of the disulfide linkage occurred within the target cell. The zwitterionic nature of NIR dyes minimizes nonspecific interactions with serum proteins, thereby enhancing the tumor-to-background signal ratio (TBR). An in vivo fluorescence imaging study demonstrated a TBR value of 2.65 within 3 h of the intravenous injection of Q-cRGD, confirming its potential utility in imaging-guided brain cancer surgery.

## 2. Materials and Methods

### 2.1. Materials

Sodium dodecyl sulfate (SDS), 2-mercaptoethanol (2-ME), dithiothreitol (DTT), and butyonine sulfoximine (BSO) were purchased from Sigma-Aldrich (St. Louis, MO, USA). Eagle’s Minimum Essential Medium (EMEM), fetal bovine serum (FBS), antibiotic/antimycotic, Hoechst33342, and Blue-DND LysoTracker were purchased from Thermo Fisher Scientific (Waltham, MA, USA). The unlabeled cRGD peptide was synthesized and purchased from FutureChem Co., Ltd. (Seoul, Republic of Korea). ATTO680-NHSester was obtained from ATTO-TEC GmbH (Siegen, Germany).

### 2.2. Synthesis and Characterization of Q-cRGD and ON-cRGD

Q-cRGD was synthesized and purchased from Peptron Inc. (Daejeon, Republic of Korea). Briefly, cystamine-W-miniPEG2-cRGDyK was synthesized using a standard 9-fluorenylmethoxycarbonyl solid-phase peptide synthesis (Fmoc-SPPS) protocol with ASP48S. It was then conjugated with the ATTO680 NHS ester dye via amide bonding to form Q-cRGD. The detailed synthetic procedures are described in the Supplementary Information. For comparison, the always-on type cRGD probe (ON-cRGD) was synthesized by conjugating Cy5.5. dye with the cRGDyK peptide (BioActs, Incheon, Republic of Korea). These products were analyzed using high-performance liquid chromatography (HPLC) and mass spectrometry (MALDI-TOF). UV–vis absorption spectra of Q-cRGD and ON-cRGD (Beckman Coulter, DU730, Brea, CA, USA) were obtained after dissolving the probes in phosphate-buffered saline (PBS, pH 7.4) and denaturing buffer (i.e., 1% SDS + 1 mM 2-ME) at a 2 μM concentration, respectively. The corresponding fluorescence spectra (ex. 620 nm, em. 650–850 nm) in PBS and denaturation buffer were measured using a microplate reader.

Next, to further verify the redox-responsive recovery of the quenched fluorescence of Q-cRGD, Q-cRGD and ON-cRGD were treated with DTT at various concentrations (0, 2 μM, and 10 mM). Fluorescence intensities were measured using a microplate reader at 2 min and every 1 h for 12 h post-treatment (ex. 625/35 nm, em. 700/20 nm).

### 2.3. In Vitro Cell Studies

#### 2.3.1. Cell Culture

MCF7 (human breast adenocarcinoma) and U87-MG (human glioblastoma) were obtained from the American Type Culture Collection (ATCC; Manassas, VA, USA). MCF7 and U87-MG cells were cultured in EMEM supplemented with 10% FBS and 1% antibiotic/antimycotic at 37 °C in a 5% CO_2_ atmosphere, respectively.

#### 2.3.2. Targeting Specificity and Fluorescence Activation in Cell

The target specificity of the synthesized probes and the fluorescence activation of Q-cRGD inside the target cancer cells were evaluated using MCF7 (low integrin receptor expression) and U87-MG (high integrin receptor expression) cancer cells. In brief, MCF and U87-MG cells were treated with Q-cRGD and ON-cRGD at a 2 μM concentration for 2 h, respectively. After washing, the cells were treated with 100 nM LysoTracker for 30 min, and confocal images were obtained using a confocal laser microscope (LSM880, AxioObserver, C-Apochromat 40×/1.2 W Korr FCS M27, Carl Zeiss, Oberkochen, BW, Germany) (cRGD probes: ex. 633 nm, em. 638–759 nm, LysoTracker, ex. 405 nm, em. 410–485 nm). Quantitative analysis of the mean fluorescence intensities per cell was performed by analyzing more than 35 cells from each group.

#### 2.3.3. Confirmation of Off-to-On Mechanism

The receptor-mediated endocytosis and subsequent redox-responsive fluorescence activation of Q-cRGD peptides were evaluated in U87-MG cells. For the competition assay for receptor binding, cells were preincubated with excess unlabeled cRGD peptides (144 μM) for 30 min and then treated with Q-cRGD (2 μM). To verify redox-responsive fluorescence activation, U87-MG cells were incubated with 0.4 mM BSO for glutathione depletion [21] for 24 h and then treated with Q-cRGD (2 μM) for 2 h. After washing, confocal images of the cells were obtained. The fluorescence intensity of the images was analyzed using Zen software 2.3 lite blue (Carl Zeiss, Oberkochen, BW, Germany).

### 2.4. In Vivo NIR Fluorescence Imaging

All of the animal experiments were approved by the Institutional Animal Care and Use Committee. Female BALB/c-nu mice (5 weeks old; Orient Bio, Seoul, Republic of Korea) were used for in vivo experiments. U87-MG cells (5 × 10^6^ cells/0.1 mL Hank’s Balanced Salt Solution) were implanted subcutaneously into the right hind flank of each mouse. The mice were divided into four groups (*n* = 3 per group) when the tumor size reached approximately 190 mm^3^. The mice in the probe treatment groups received intravenous injections of Q-cRGD and ON-cRGD (20 nmol/100 µL/mouse), respectively. Mice in the competition assay group received an intravenous injection of Q-cRGD (20 nmol) mixed with unlabeled cRGD (400 nmol). The mice in the control group received PBS solution (100 µL/mouse). Optical images were obtained using the IVIS Lumina imaging system (ex. = 660/20 nm, em. = 710/40 nm) at 10 min and 3 h post injection. For comparison, in vivo fluorescence imaging was conducted 24 h post injection in the ON-cRGD-treated group. TBR values were analyzed from the fluorescence images obtained at each time point. The fluorescence signal originating from the dorsal side of the mouse was used as the background.

Another set of mice was used for the biodistribution study of Q-cRGD and ON-cRGD. Three hours post-injection, tumors and major organs (spleens, kidneys, and livers) were collected, and ex vivo fluorescence images of these tissues were obtained. Then, the tumors were immediately frozen and cryo-sectioned at a 7 μm thickness. The nuclei of the tumor cells were counter-stained with Hoechst33342. Fluorescence images of tumor sections (cRGD probes, ex. 624–674 nm, em. 689–729 nm, Nuclei: ex. 375–407 nm, em. 438–468 nm) were obtained using a Vectra Polaris (Akoya Biosciences, Marlborough, MA, USA).

### 2.5. Statistical Analysis

Student’s *t*-test was used to determine significant differences between the test groups. Data are expressed as mean ± S.D.

## 3. Results and Discussion

### 3.1. Synthesis of Q-cRGD and ON-cRGD

The chemical structures of the probes used in this study are shown in Figure 1B. Two fluorescent dyes were used in this study. The Q-cRGD agent comprises a cRGD peptide linked by short ethylene glycol to tryptophan and a glutathione-cleavable, disulfide-bonded zwitterionic ATTO 680 dye. ON-cRGD served as a control agent, with the cRGD peptide being conjugated to Cy5.5 via a short alkyl-amide chain. The formation of Q-cRGD and ON-cRGD was verified using HPLC and MALDI-TOF, as illustrated in Figures S1 and S2, respectively. The molecular weights of Q- and ON-cRGD were 1635.67 and 1516.73 g/mol, respectively.

### 3.2. Spectral Properties of Q-cRGD and ON-cRGD

**Figure 2:**
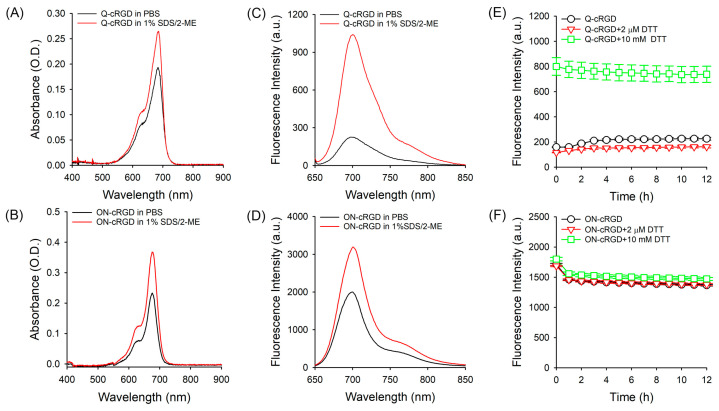
Optical properties of Q- and ON-cRGD probes. UV–vis spectrum of (**A**) Q-cRGD and (**B**) ON-cRGD in different buffer solutions (concentration: 2 μM dye equivalent). Corresponding fluorescence spectra of (**C**) Q-cRGD and (**D**) ON-cRGD in different buffer solutions (ex. 620 nm, em. 650–850 nm). Changes in fluorescence intensity of (**E**) Q-cRGD and (**F**) ON-cRGD after treatment with DTT. DTT was applied at concentrations of 0 and 2 μM and 10 mM, and their fluorescence intensities were measured using microplate reader at 2 min and every 1 h for 12 h post-treatment (ex. 625/35 nm, em. 700/20 nm).

To validate our design concept, we recorded the UV–vis absorption and fluorescence spectra. Q-cRGD exhibited an absorption maximum at approximately 690 nm in PBS (Figure 2). The addition of SDS and 2-ME, the two denaturing agents, resulted in increased absorption with a slight hypsochromic shift. Similarly, the ON-cRGD peptide showed increased absorption upon the addition of SDS and 2-ME (Figure 2A,B). Interestingly, the addition of SDS and 2-ME to Q-cRGD led to a seven-fold increase in fluorescence at 700 nm, whereas ON-cRGD exhibited a 1.5-fold increase at the same wavelength (Figure 2C,D). Because ON-cRGD has no quenching moiety, the increase in the fluorescence intensity of ON-cRGD seems to be due to the change in its absorption spectrum after the addition of the SDS surfactant. The significant increase in the fluorescence intensity of Q-cRGD was due to the cleavage of disulfide bonds by 2-ME, the release of ATTO680 dye from Trp, and the subsequent disappearance of the PET quenching effect. The fluorescence quenching of the ATTO680 dye by tryptophan in close proximity via the PET mechanism has been studied in detail in previous reports [10,22].

As mentioned above, 2-ME cleaves disulfide bonds, thereby alleviating quenching from neighboring Trp residues. To further substantiate this, we added the reducing agent dithiothreitol (DTT) to the Q- and ON-cRGD probes. As shown in Figure 2E, the fluorescence intensity at 700 nm did not increase with 2 μM of DTT but surged by 4.5-fold right after the addition of 10 mM of DTT (Figure 2E). This indicates that the cleavage of the disulfide bond relieved the ATTO 680 dye from its quenched state, restoring its fluorescence. By contrast, ON-cRGD treated with 10 mM DTT did not exhibit any increase in fluorescence at 700 nm (Figure 2F). These data also support the idea that the increase in the fluorescence intensity of ON-cRGD shown in Figure 2D was due to the effect of the SDS surfactant. These findings demonstrate that the quenched fluorescence of Q-cRGD can be activated in response to reducing agents inside the target cells after entering the cells via receptor-mediated endocytosis.

### 3.3. Targeting Specificity and Fluorescence Activation in Cell

#### 3.3.1. In Vitro Fluorescence Imaging of MCF-7 and U87-MG Cell Lines

**Figure 3:**
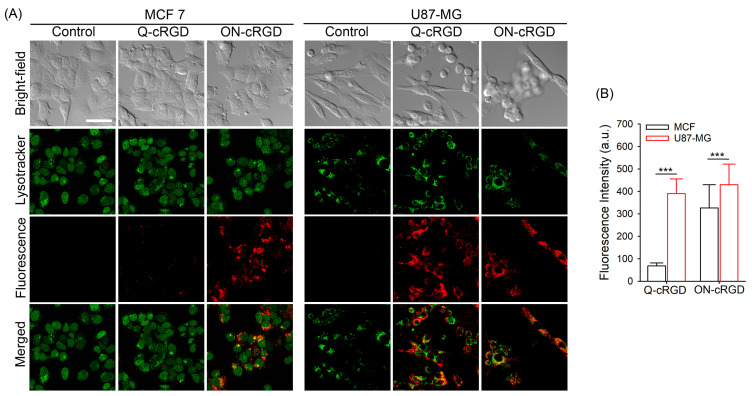
An evaluation of the target specificity of Q- and ON-cRGD probes. (**A**) Confocal images of the Q- and ON-cRGD-treated cancer cells. The MCF 7 (integrin receptor low expressed) and U87-MG (integrin receptor high expressed) cells were treated with Q- and ON-cRGD probes at 2 μM for 2 h. After adding 100 nM of lysotracker, these cells were washed, and confocal images were obtained (ATTO680; ex. 633 nm, em. 638–759 nm, Lysotracker; ex. 405 nm, em. 410–485 nm). Scale bar = 50 μm. (**B**) A comparison of fluorescence intensities in the Q- and ON-cRGD-treated MCF7 and U87-MG Cells. A quantitative analysis of the mean fluorescence intensities per cell was performed by analyzing more than 35 cells from each group. *** *p* < 0.001.

To demonstrate the advantage of activation, we compared the in vitro imaging qualities of two cell lines with differing levels of integrin expression (Figure S3). The breast cancer cell line MCF-7, which has low integrin expression, and the human glioblastoma cell line U87-MG, which has high integrin expression, were treated with Q- and ON-cRGD probes (Figure 3A). Both cell lines were incubated with 2 μM probes and then costained with LysoTracker. Both Q-cRGD- and ON-cRGD-stained U87 cells exhibited strong fluorescence signals with an overlap between the signals of LysoTracker and the dye, as shown in Figure 3A. In the case of the Q-cRGD-treated MCF-7 cells, weak fluorescence signals were observed in the probe. However, the ON-cRGD-treated MCF-7 cells exhibited high fluorescence intensity. The mean fluorescence intensity of the Q-cRGD-treated U87-MG cells was 5.7 times higher (*p* < 0.001), and that of the ON-cRGD-treated U87-MG cells was 1.3 times higher (*p* < 0.001) than that of their respective Q-cRGD- and ON-cRGD-treated MCF7 cells (Figure 3B). Considering the mean fluorescence intensity in MCF7 cells that was 8.7 times lower than that of the U87-MG cells according to the flow cytometric analysis, the relatively high fluorescence in the ON-cRGD-treated MCF-7 cells indicated nonspecific uptake into cancer cells expressing low integrin levels. When a competition assay for the receptor binding of ON-cRGD was performed in the MCF7 cells, pretreatment with excess cRGD peptides did not reduce the fluorescence intensity in the ON-cRGD-treated cells (Figure S4). This suggests that the high uptake of ON-cRGD in MCF7 cells is nonspecific. These data support that the target specificity of the Q-cRGD probe in response to integrin expression levels is much better than that of the always-on type of ON-cRGD probe and thus has high potential to serve as an optical agent for intraoperative guidance. Since the fluorescence of Q-cRGD is quenched in its native state, strong fluorescence signals inside the Q-cRGD-treated U87 cells indicate fluorescence recovery inside the cells after receptor-mediated endocytosis. As mentioned above, zwitterionic dyes are beneficial for avoiding nonspecific binding to serum proteins. Because the molecular size of the cRGD-fluorophore conjugate (~1500 g/mol) is 44 times smaller than that of bovine serum albumin (66,431 g/mol) [23], unwanted binding of the conjugated dye with serum proteins (either by charge–charge interactions or by binding to the hydrophobic pocket in albumins) can affect the target specificity of cRGD-fluorophore conjugates. The small difference in fluorescence signals between the ON-cRGD-treated MCF7 and U87-MG cells indicated that the binding of the negatively charged Cy5.5 dye hampered the target specificity of Q-cRGD.

#### 3.3.2. Confirmation of Off-to-On Mechanism

**Figure 4:**
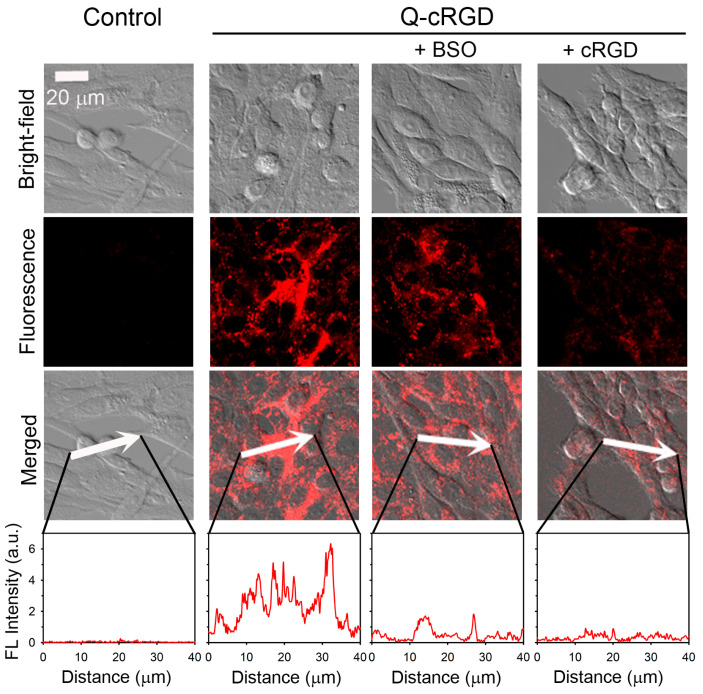
Evaluation of redox-responsive fluorescence activation of Q-cRGD after receptor-mediated endocytosis into U87-MG cells. For inhibition of glutathione synthesis, cells were pretreated with 0.4 mM buthionine sulfoximine (BSO) for 24 h and incubated with 2 μM Q-cRGD. For competition assay of receptor binding, cells were pretreated with excess cRGD peptides (7.2 nmol) for 30 min, and then 2 μM Q-cRGD was added for 2 h (ex. 633 nm, em. 638–758 nm). Scale bar = 20 μm. Fluorescence intensity across area indicated by white arrows (third panel) was analyzed and displayed in bottom panels.

To further investigate the mechanism underlying Q-cRGD activation, we either blocked integrin receptors with free RGD peptides or reduced the glutathione levels by treating the cells with BSO. As shown in Figure 4, the U87-MG cells treated with 0.4 mM of BSO exhibited a much weaker fluorescence signal compared to the untreated cells. In addition, the U87-MG cells treated with excess free cRGD peptides (144 μM) showed an even weaker fluorescence signal than those treated with BSO. A quantitative linear scan obtained using a confocal microscope is provided in Figure 4 (bottom panel). These results indicate that the Q-cRGD probes are taken up by cells through integrin recognition on the cell surface and are subsequently processed in the endosomal pathway by glutathione, which alleviates the quenching effect of the tryptophan-based PET mechanism.

### 3.4. In Vivo Imaging of U87-Xenografted Mice Model

**Figure 5:**
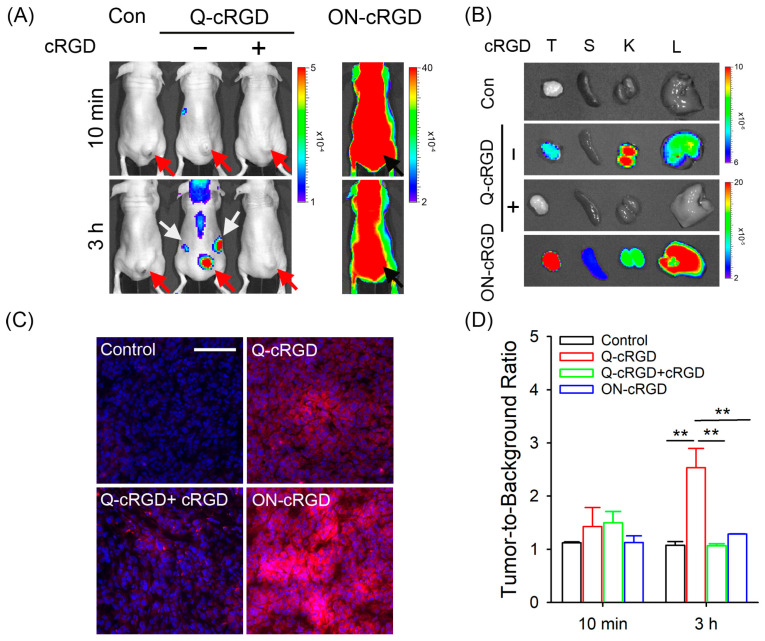
In vivo evaluation of Q-cRGD in U87-MG xenograft tumor model. (**A**) NIR fluorescence images of Q-cRGD, ON-cRGD, and Q-cRGD+cRGD-treated mice. Q-cRGD and ON-cRGD (20 nmol/100 μL) were intravenously injected via tail vein, and images were obtained with time using IVIS systems (ex. 660/20 nm, em. 710/40 nm). For competition binding assay, 400 nmol cRGD was mixed with 20 nmol Q-cRGD in 100 μL and intravenously injected. Red and black arrows: tumor sites; white arrows: fluorescence from kidneys. (**B**) Ex vivo images of tumors and major organs obtained at 3 h post-injection. (**C**) Analysis of NIR fluorescence signals in tumor sections. Red: fluorescence from injected probes; blue: nuclei. Scale bar = 100 μm. (**D**) Comparison of tumor-to-background ratio in each group. ** *p* < 0.01.

BALB/c-nu mice implanted with U87-MG cancer cells were used to demonstrate the potential utility of Q-cRGD in tumor-imaging-guided brain surgery (Figure 5 and Figure S5). The Q- and ON-cRGD probes were administered via tail vein injection. In vivo NIR fluorescence images of the Q-cRGD-treated mice show minor fluorescence from the body at 10 min, whereas strong fluorescence signals were detected in the tumors and kidneys at 3 h post-injection (Figure 5A). Cotreatment with excess cRGD peptides resulted in no fluorescence signals in the tumors and kidneys at 3 h post-injection. Figure 5B shows the ex vivo fluorescence images of the tumors and major organs collected 3 h post-injection. Strong fluorescence was observed in the tumors. Notable fluorescence in the liver is due to integrin expression in hepatocytes [24]. Strong fluorescence intensity in the kidneys indicated that some of the cleaved ATTO680 dyes inside the integrin-expressing tumor cells and hepatocytes were secreted from the cells and primarily cleared through renal excretion. The renal excretion of intravenously injected dyes is a representative feature of zwitterionic dyes [3,19]. Interestingly, no fluorescence signals were observed in the tumors, kidneys, or livers of the group cotreated with excess cRGD. These data indicate that excess cRGD peptides prevented the binding of Q-cRGD probes to integrin-overexpressing tumor cells and hepatocytes. An analysis of the tumor tissue cross-sections (Figure 5C) also supported the receptor-mediated uptake of Q-cRGD and subsequent fluorescence activation inside the tumor cells. No signals in the kidneys in the cotreatment group of the Q-cRGD-treated mice were observed because the injected Q-cRGD remained in a quenched state in the blood circulation or during renal excretion. Overall, these data confirm that the Q-cRGD probe remains in a quenched state during blood circulation, and its fluorescence is specifically turned on inside integrin-overexpressing tumors after receptor-mediated endocytosis and the subsequent release of the conjugated dyes. For intraoperative guidance, effectiveness within the surgical window, usually within 4 h, is crucial [15]. Compared with ON-cRGD, the Q-cRGD probes achieved a high TBR (2.65) at 3 h post-injection, making them suitable for use as intraoperative optical probes.

In this study, mice treated with ON-cRGD exhibited strong fluorescence signals from the tumor and throughout the body at 10 min post-injection, maintaining these signals until 3 h post-injection (Figure 5A and Figure S5B). As a result, the TBR values in the ON-cRGD-treated mice were 1.29 and 2.03 at 3 and 24 h post-injection, respectively (Figure S5C). Chen et al. reported that when mice were administered 3 nmol of ON-cRGD, the TBR at 4 h post-injection was also below 2 [13]. Meanwhile, previous studies have demonstrated that an injection of 0.5 nmol of ON-cRGD can achieve a high TBR close to 3 within the same timeframe [8,13]. Brain tumors embedded within normal brain tissues present unique detection challenges. In such cases, a strong fluorescence signal at tumor sites, coupled with a TBR, is crucial for identifying brain tumors obscured by surrounding normal tissue. Therefore, since administering a low dose of ON-cRGD reduces the fluorescence intensity at tumor sites, further studies are necessary to determine whether brain tumors can be detected in vivo with this optimal dose (i.e., ≤1 nmol) of ON-cRGD.

## 4. Conclusions

In this study, we developed a zwitterionic Q-cRGD probe for efficient brain tumor imaging. After RGD-integrin recognition, the probe is endocytosed into the cell and the dye is cleaved from the tryptophan quencher by the intracellular-reducing agent glutathione. A high tumor-to-background ratio was achieved in the Q-cRGD-treated mice 3 h post-injection. These results suggest that the Q-cRGD probe is a promising candidate for imaging-guided brain tumor surgery.

## Data Availability

The datasets generated and/or analyzed during the current study are available from the corresponding author upon reasonable request.

## Supplementary Materials

The following supporting information can be downloaded at https://www.mdpi.com/article/10.3390/pharmaceutics16081034/s1, Figure S1: HPLC and MALDI-TOF data of Q-cRGD; Figure S2: HPLC and MALDI-TOF data of ON-cRGD; Figure S3: Flow cytometric analysis of integrin expression in different cell lines; Figure S4: Competition assay for receptor binding of ON-cRGD in MCF7 cells; Figure S5: Optical images of Q-cRGD- and ON-cRGD-treated mice.
