# Supplementary material for: Quenched Zwitterionic Cyclic Arg-Gly-Asp-Containing Pentapeptide Probe for Real-Time Brain Tumor Imaging
Source: Pharmaceutics. 2024 Aug 2;16(8):1034. doi: 10.3390/pharmaceutics16081034 (PMC11360280; doi:10.3390/pharmaceutics16081034)
Supplement: Supplementary file 1 [file pharmaceutics-16-01034-s001.zip › pharmaceutics-3124020-supplementary.pdf]

Supplementary Information

Quenched zwitterionic cRGD probe for real-time brain tumor imaging

Hyunjin Kim,[a†] Maixian Liu,[a†] and Yongdoo Choi*[a]

[a]Division of Technology Convergence, National Cancer Center, 323 Ilsan-ro, Goyang, Gyeonggi-Do, 10408, Republic of Korea.

†Hyunjin Kim and Maixian Liu contribute equally to this work

*Corresponding author. E-mail:ydchoi@ncc.re.kr

Materials

ATTO680-NHSester was obtained from ATTO-TEC GmbH (Siegen, Germany). Deprotected cRGDyK for the competition assay and RGDyK-Cy5.5 (ON-cRGD) were synthesized and purchased from FutureChem Co., Ltd. (Seoul, Republic of Korea) and Bioacts (Incheon, Republic of Korea), respectively. Trt-Cl resin and Fmoc amino acids were purchased from GL BioChem (Shanghai, China). Coupling and cleavage cocktail reagents were purchased from Sigma Aldrich, and other solvents were purchased from Daejung Chemical (Dasan, Republic of Korea).

Methods

General procedure Cystamine-CW-miniPEG2-cRGDyK peptide synthesis

Cystamine-CW-miniPEG2-cRGDyK was synthesized using standard Fmoc solid-phase peptide synthesis (SPPS); Fmoc-Asp-OAll was loaded onto Trt resin, Fmoc protecting group was removed by rocking in 20% piperidine in DMF for 10 min (twice), and serial coupling was performed using Fmoc amino acids (8 equiv), HOBT (8 equiv), HBTU (8 equiv), DIPEA (16 equiv.) in DMF for 2 hrs. Fmoc-Lys (Dde)-OH was used for lysine side chain elongation. When the desired linear sequence was completed, the allyl group was removed using tetrakispalladium, followed by head-to-tail cyclization using DIC. The crude peptide was cleaved from the resin using a mixture of TFA/EDT/thioanisole/TIS/DW (90/2.5/2.5/2.5/2.5 Volume) for 2 h. The solution was precipitated with cold ether and pelleted via centrifugation. The solids were collected and air-dried. Aldrithiol was used for thiol protection, and cysteamide was added to form a heterodisulfide bond. The crude peptide was dissolved in DW and purified via reverse-phase HPLC using a C18 reverse-phase column. Elution was performed using a water–acetonitrile linear gradient (10%–75% (v/v) of acetonitrile) containing 0.1% (v/v) trifluoroacetic acid. The pure peptide was collected and lyophilized.

Preparation of Q-cRGD probe

Cystamine-CW-miniPEG2-cRGDyK (1.98 μmole) and ATTO680-NHSester (2.97 μmole) were reacted in 0.5 mL PBS buffer by stirring for 1 h. The reaction was stopped using 20% glycerol, then identified the peaks by RP-HPLC, and collected the final products using prep-HPLC (Alliance HPLC system, Waters Corporation, MA. USA.).

Flow cytometric analysis

To identify integrin expression in MCF7 and U87-MG cancer cells, cells were seeded into a 6-well plate and treated with AngioFlamma 675 (Bioacts, Republic of Korea) for 2 hours at a concentration of 1 μM. Subsequently, the cells were detached and washed three times with PBS buffer. Finally, the cells were resuspended in PBS buffer and analyzed using a flow cytometer (LSRFortessa, BD Bioscience; ex. 640 nm, em. 730/45 nm).

Figures

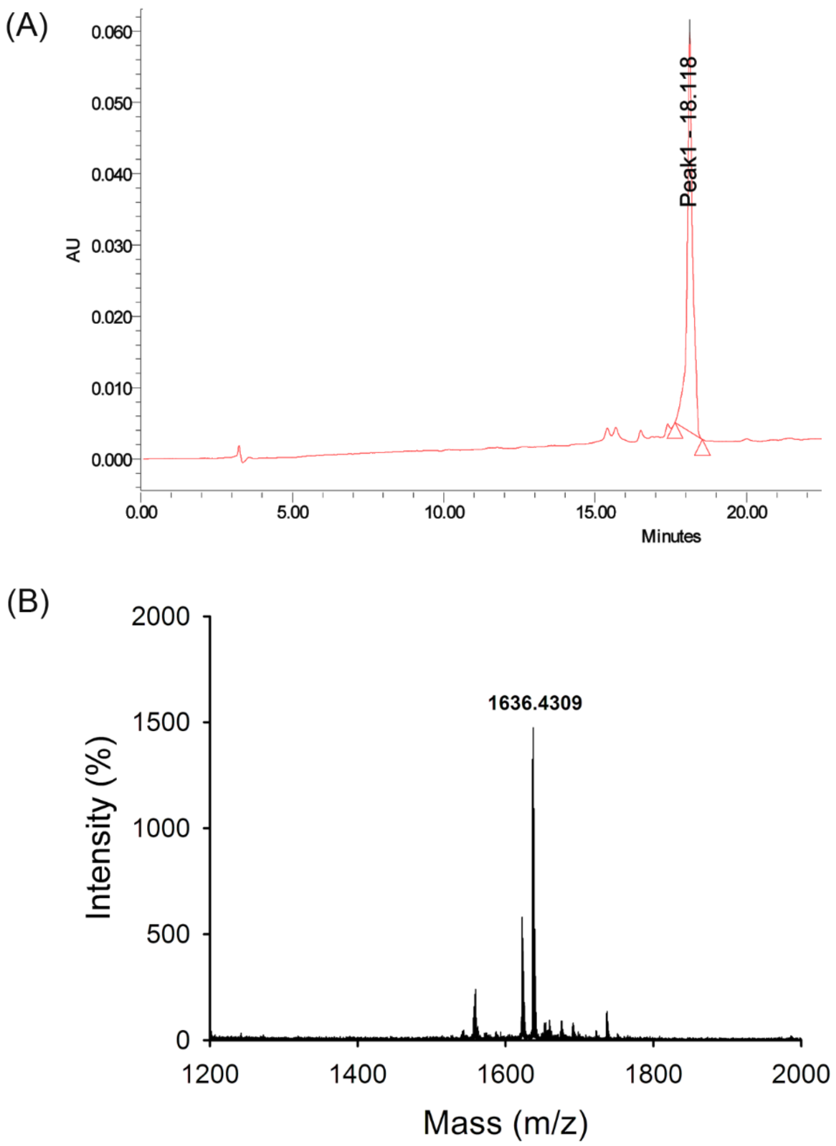

Figure S1. Characterization of Q-cRGD. (A) HPLC chromatogram and (B) MALDI-TOF spectrum of Q-cRGD

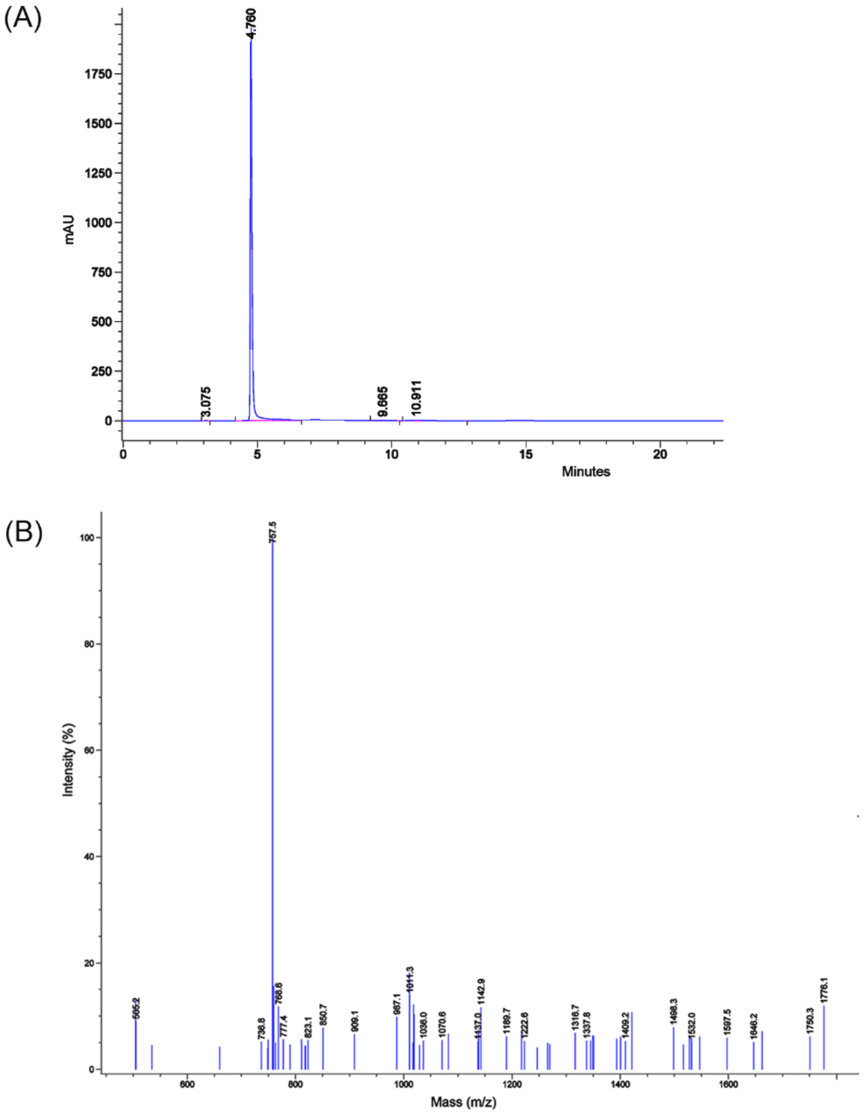

Figure S2. Characterization of ON-cRGD. (A) HPLC chromatogram and (B) MALDI-TOF spectrum of ON-cRGD

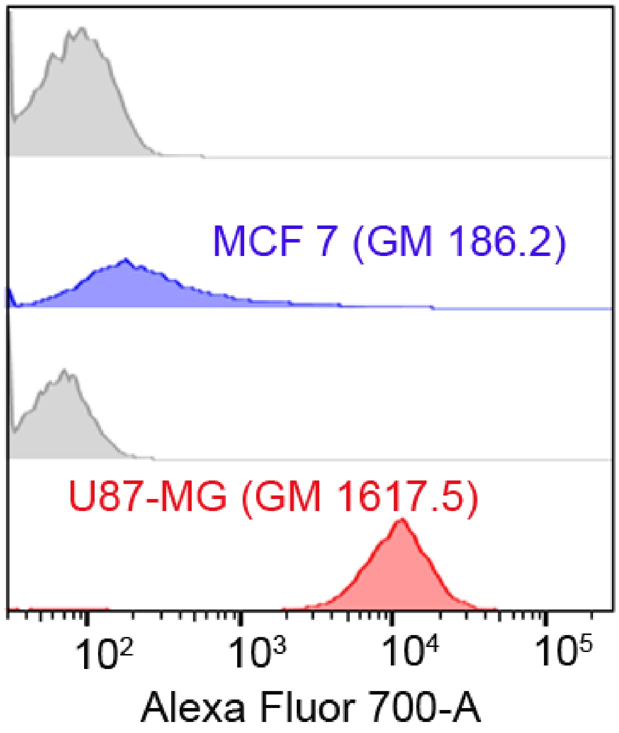

Figure S3. Flow cytometric analysis of integrin expression in MCF7 and U87-MG cancer cells (ex. 640 nm, em. 730/45 nm). These cells were treated with AngioFlamma 675 (Bioacts) for 2 h at the concentration of 1 μM and flow cytometry analysis was conducted.

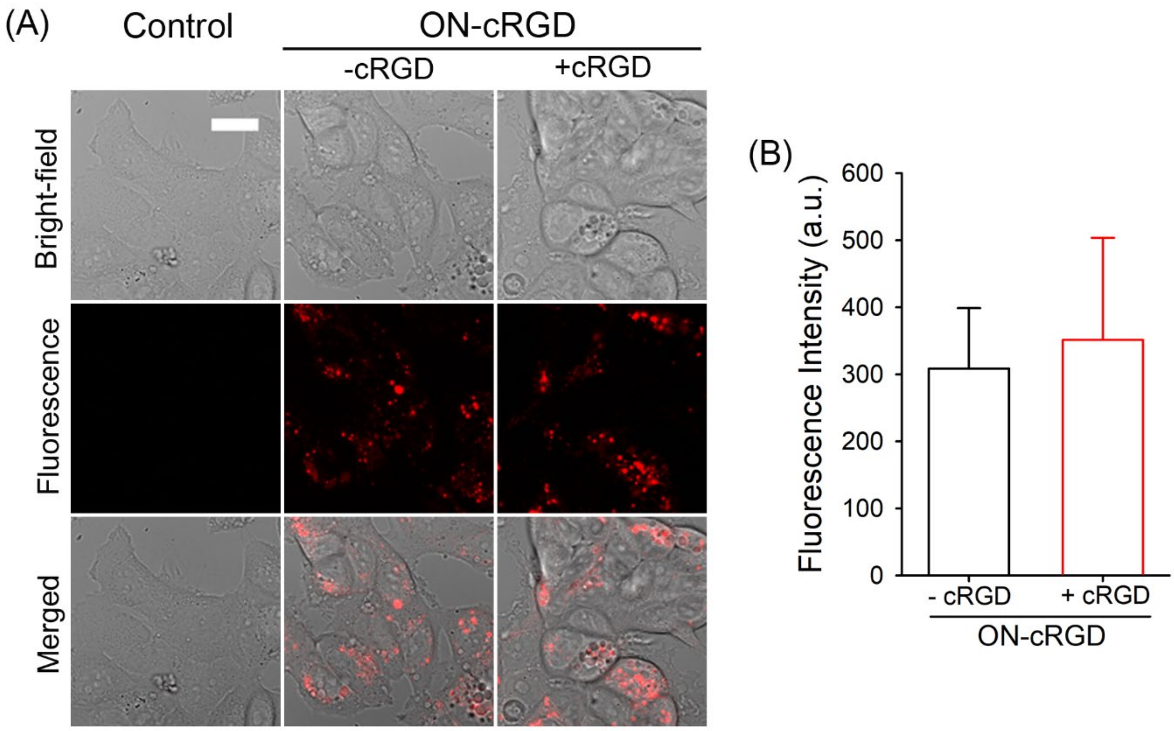

Figure S4. Competition Assay for Receptor Binding of ON-cRGD in MCF7 Cells. (A) Confocal images of ON-cRGD-treated MCF7 cells, both with and without pretreatment with excess cRGD peptide. Scale bar = 20 μm. (B) Comparison of Fluorescence Intensities in ON-cRGD-Treated MCF7 Cells. Low integrin expressin MCF cells were preincubated with excess unlabeled cRGD peptide (144 μM) for 30 min and then treated with ON-cRGD (2 μM). After washing, confocal images of the cells were obtained. The fluorescence intensity of the images was analyzed using Zen software (Carl Zeiss, Germany).

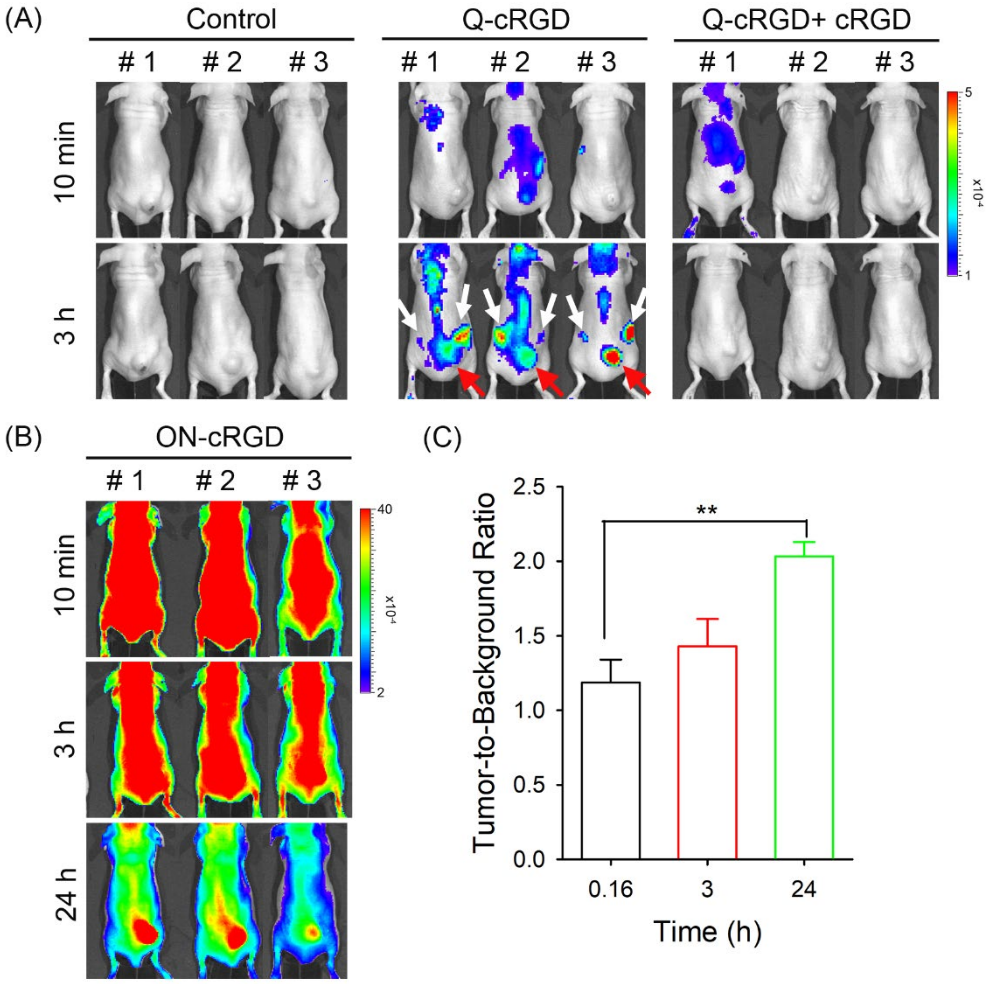

Figure S5. (A) NIR fluorescence images of control, Q-cRGD, and Q-cRGD+cRGD treated mice. (n=3). (B) NIR fluorescence images of ON-cRGD treated mice (n=3). (C) Tumor-to-Background Ratios in ON-cRGD-treated mice over time (n=3). ***P*<0.01.
